# Direct repression of the oncogene CDK4 by the tumor suppressor miR-486-5p in non-small cell lung cancer

**DOI:** 10.18632/oncotarget.8514

**Published:** 2016-03-31

**Authors:** Yang Shao, Yu-Qing Shen, Yan-Li Li, Chen Liang, Bing-Jie Zhang, Sheng-Di Lu, Yan-Yun He, Ping Wang, Qiang-Ling Sun, You-Xin Jin, Zhong-Liang Ma

**Affiliations:** ^1^ School of Life Sciences, Shanghai University, Shanghai, China; ^2^ Shanghai Jiao Tong University Affiliated Sixth People's Hospital, Shanghai, China; ^3^ Central Laboratory, Shanghai Chest Hospital, Shanghai Jiaotong University, Shanghai, China; ^4^ Experimental Center for Life Sciences, Shanghai University, Shanghai, China

**Keywords:** miR-486-5p, CDK4, NSCLC, methylation, cell cycle

## Abstract

MicroRNAs are a class of non-coding single-stranded RNA, 20-23 nucleotide in length, which can be involved in the regulation of gene expression. Through binding with 3′-untranslated regions (3′-UTR), microRNAs can cause degradation of target mRNAs or inhibition of translation, and thus regulating the expression of genes at the post-transcriptional level. In this study, we found that miR-486-5p was significantly downregulated in non-small cell lung cancer (NSCLC) tissues and cell lines, suggesting that miR-486-5p might function as a tumor suppressor in lung cancer. Additionally, we showed that CDK4, an oncogene that plays an important role in cell cycle G1/S phase progression, was directly targeted by miR-486-5p. Furthermore, our data reveals that knockdown of CDK4 by siRNA can inhibit cell proliferation, promote apoptosis, and impede cell-cycle progression. In epigenetics, the upstream promoter of miR-486-5p was strongly regulated by methylation in NSCLC. Collectively, our results suggest that miR-486-5p could not only inhibit NSCLC by downregulating the expression of CDK4, but also be as a promising and potent therapy in the near future.

## INTRODUCTION

Lung cancer is the leading cause of cancer-related mortality worldwide, with non-small cell lung cancer (NSCLC) accounting for nearly 80% of all cases [1, 2]. Although improvements in early diagnosis and clinical treatment strategies have been made, the overall 5-year survival for NSCLC patients is still low (15%) and the recurrence rate remains high [3], the need to elucidate the potential mechanism for the initiation and progression of NSCLC is urgent. Future directives are aimed at further understanding these mechanisms involved in the tumorigenesis of NSCLC and prognostication on potential therapeutic targets.

MicroRNAs (miRNA) are small non-coding, endogenous, 20-23 nt [4], single-stranded RNAs that regulate gene expression by binding to the 3′-untranslated region (3′-UTR) of mRNA [5]. A myriad of important biological processes are profoundly influenced by miRNAs, including growth, differentiation, apoptosis, motility, and malignant transformation [6, 7]. In recent years, miRNAs have received great attention in cancer research, especially in NSCLC. Several deregulated miRNAs in NSCLC such as miR-34a, the let-7 family, miR-143, and miR-101 have been shown to regulate cell growth, apoptosis, migration, and invasion [8-12]. Our lab has verified that miR-34a, miR-181a-5p, and miR-32 play important roles in cancer development [8, 13, 14]. These findings indicate that altered miRNA expression may be associated with the tumorigenesis of NSCLC.

The downregulation of the miRNA, miR-486-5p, has been linked to a number of cancers and diseases [15-18]. MiR-486-5p is significantly downregulated in NSCLC [19], breast cancer [20], hepatocellular carcinoma [21], chronic kidney disease [22], spinal cord injury [23], and Duchenne muscular dystrophy (DMD) [24]. Based on this correlation, we hypothesized that miR-486-5p may play an important role in tumorigenesis and tumor development in NSCLC.

In the present study, we investigated the potential function of miR-486-5p in the development and progression of NSCLC. Not surprisingly, we found that miR-486-5p was downregulated in the majority of NSCLC patient samples and in several NSCLC cell lines. Furthermore, data from assays revealed that miR-486-5p significantly suppressed NSCLC tumor growth and cell cycle by targeting CDK4. Our results present a new potential direction for therapeutic intervention in the treatment of NSCLC through understanding of the biological effects of miR-486-5p.

## RESULTS

### MiR-486-5p is downregulated in NSCLC

**Figure 1:**
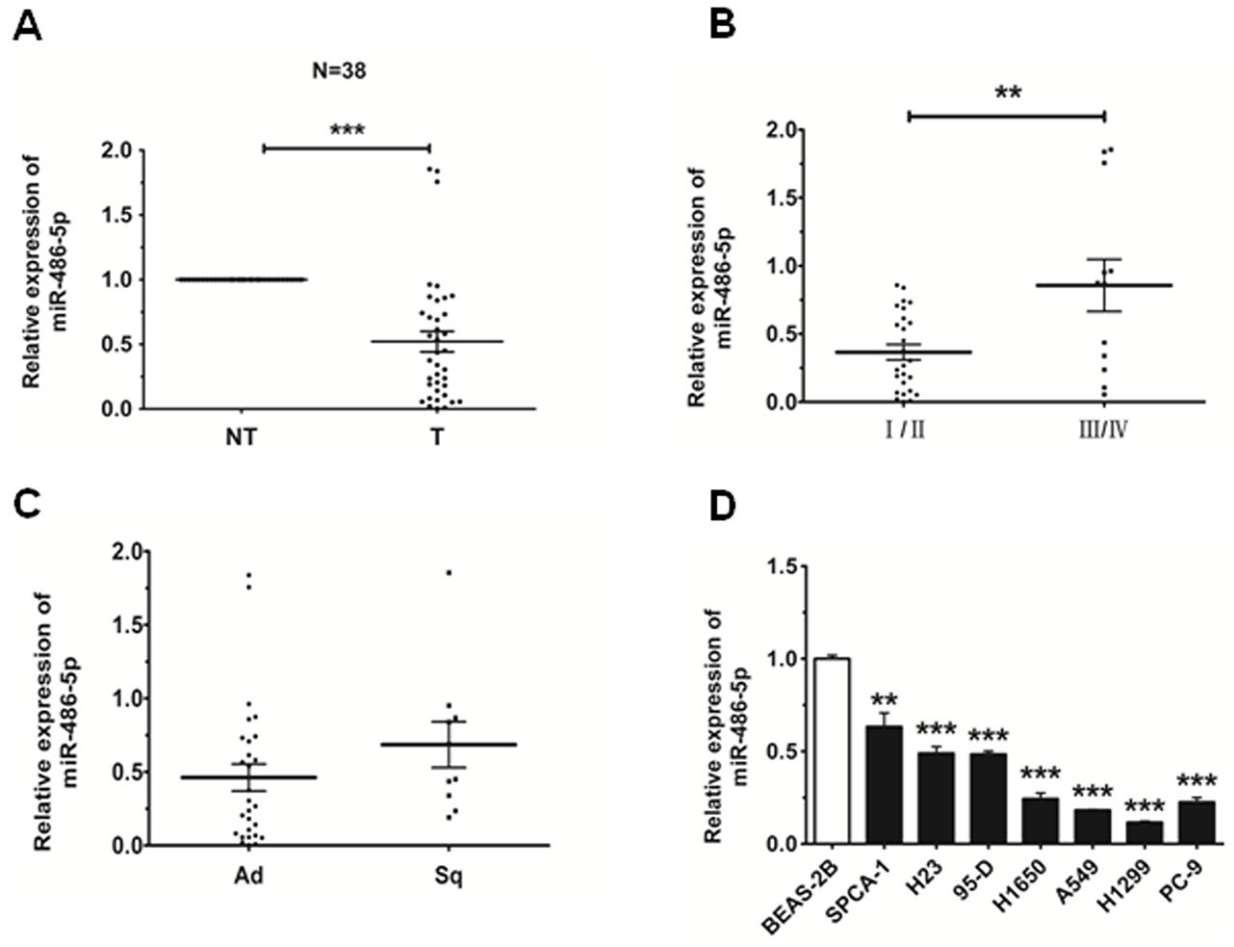
MiR-486-5p is downregulated in NSCLC tissues and cell lines **A.** The relative expression level of miR-486-5p in corresponding non-tumor tissues (NT) and tumor tissues (T) was from qRT-PCR results. U6 was used for normalization. **B.** The relative expression level of miR-486-5p in stage I/II tumors and stage III/IV tumors. **C.** The relative expression level of miR-486-5p in adenocarcinoma (Ad) and squamous cell carcinoma (Sq). **D.** The relative expression level of miR-486-5p in lung cancer cell lines or a pulmonary epithelial cell line (control) was measured by qRT-PCR. * P<0.05, ** P<0.01, *** P<0.001.

In order to discuss the role of miR-486-5p in lung carcinogenesis, we first sought to detect the expression level of miR-486-5p in 38 NSCLC patient cases. The data we had obtained revealed that miR-486-5p was downregulated in 35 cases (92%) when compared with corresponding non-tumor lung tissues (Figure 1A). Then we observed that the level of miR-486-5p was much lower in stage I/II than that in stage III/IV and showed no significant difference in adenocarcinoma (Ad) and squamous cell carcinoma (Sq, Figure 1B and 1C). The expression of miR-486-5p in NSCLC cell lines was also examined, and there we found that miR-486-5p was significantly downregulated in SPCA-1 (P<0.01), 95-D, H1650, A549, H1299, PC-9, and H23 cells (p<0.001), as compared with BEAS-2B control cells (Figure 1D). These results suggested that the reduction in miR-486-5p expression was associated with NSCLC carcinogenesis. Based on this finding, the H1299 and SPCA-1 cell lines were chosen to test the effects of miR-486-5p on cell proliferation and cell cycle.

### MiR-486-5p inhibits cell proliferation and impedes cell cycle progression in NSCLC

**Figure 2:**
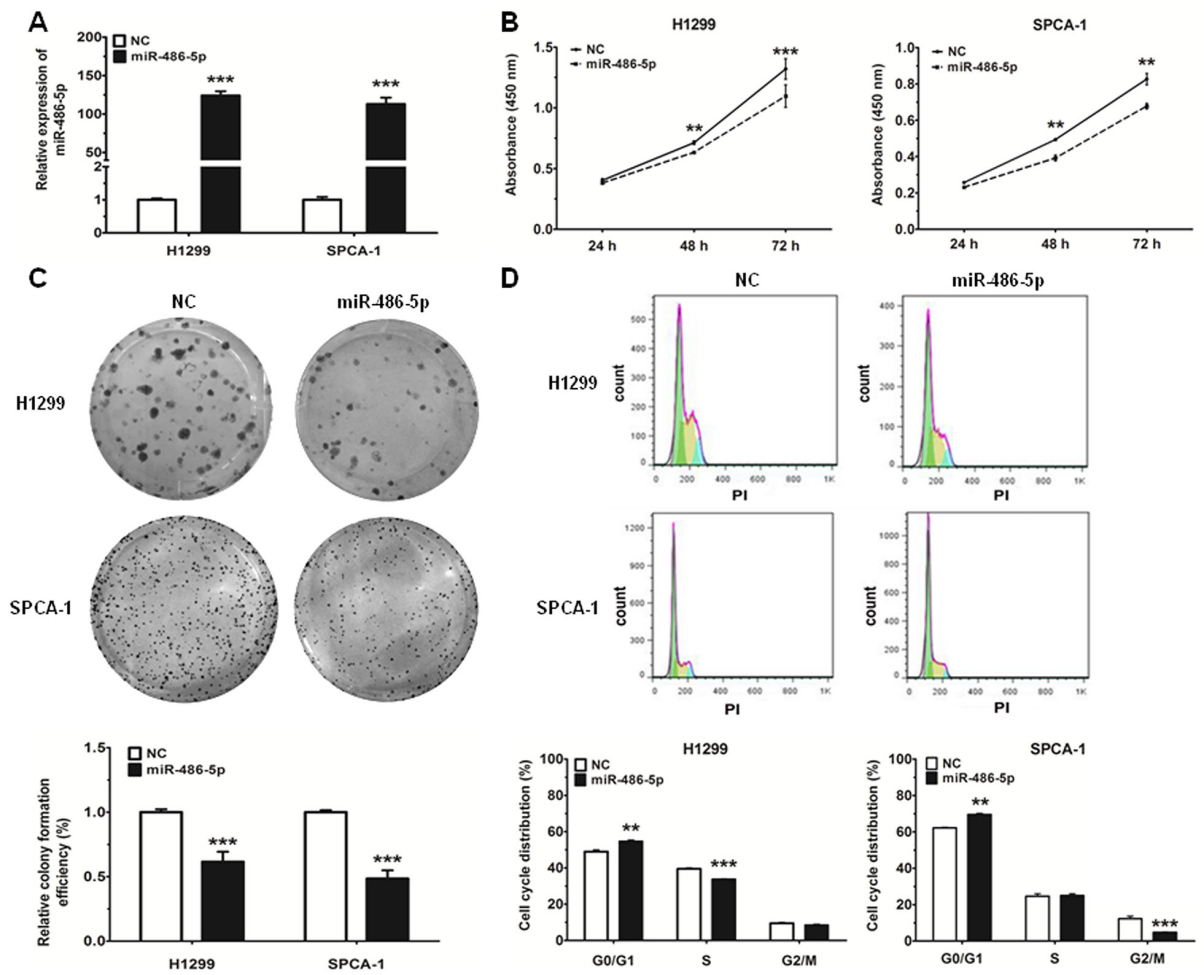
MiR-486-5p can inhibit cell proliferation and impede cell-cycle progression in NSCLC cell lines **A.** The expression of miR-486-5p was measured by qRT-PCR in H1299 and SPCA-1 cells 24 hours after transfection with negative control (NC) and miR-486-5p mimics. **B.** H1299 and SPCA-1 cells were transfected with NC or miR-486-5p mimic, and cell proliferation was determined by CCK-8. **C.** Colony formation assay in H1299 and SPCA-1 cells transfected with NC or miR-486-5p mimic. **D.** The cell cycle distributions of H1299 and SPCA-1 cells transfected with NC or miR-486-5p mimic were detected by flow cytometry. ** P<0.01, *** P<0.001.

In order to investigate the effect of miR-486-5p on NSCLC cell proliferation, H1299 and SPCA-1 cells were transfected with miR-486-5p mimic, and the results of qRT-PCR analysis validated that transfection of miR-486-5p did increase its level in both cell lines (Figure 2A). Proliferation of NSCLC cells was assessed by CCK-8 assays and cell colony assays (Figure 2B and 2C). Our results showed that cellular proliferation gradually declined following transfection with miR-486-5p mimic in both H1299 and SPCA-1 cells. Treatment of cells with miR-486-5p mimic led to a significant decrease in NSCLC cell growth after 72 h, when compared with the negative control (NC). Additionally, 48 h after transfection with miR-486-5p mimic, the proportion of cells at G0/G1 phase increased more than 5% over the control (Figure 2D). These results demonstrated that miR-486-5p could inhibit the proliferation of NSCLC cells and cause G0/G1 cell-cycle arrest.

### MiR-486-5p targets the 3′-UTR of CDK4 directly

**Figure 3:**
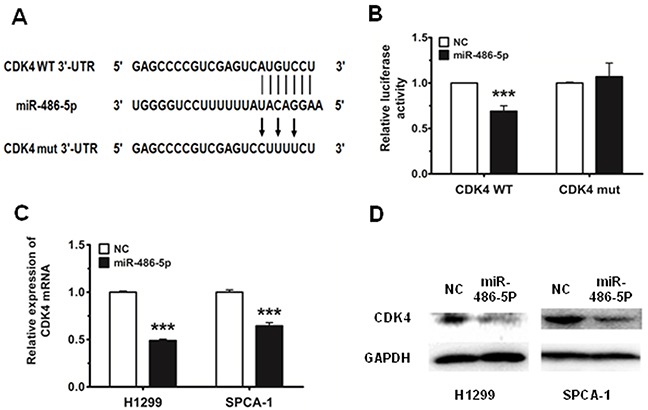
CDK4 is a direct target of miR-486-5p **A.** CDK4 WT 3′-UTR contains predicted miR-486-5p binding sites. The figure shows alignment of miR-486-5p with CDK4 WT 3′-UTR and the arrows indicate the mutagenesis nucleotides. **B.** Dual luciferase reporter assay. Luciferase reporter constructs containing pGL3-CDK4 WT 3′-UTR (CDK4 WT) and pGL3-CDK4 mut 3′-UTR (CDK4 mut) were co-transfected with miR-486-5p mimic or NC mimic in HEK293T cells. Relative firefly luciferase expression is displayed, normalized to Renilla luciferase expression. **C.** and **D.** The mRNA levels of CDK4 were measured by qRT-PCR and the protein levels were measured by western blot in H1299 and SPCA-1 cells transfected with NC and miR-486-5p mimic. *** P<0.001.

Using in TargetScan prediction programs (www.targetscan.org), we identified CDK4 as a potential target for miR-486-5p. To verify whether CDK4 is a direct target of miR-486-5p, the CDK4 wild type 3′-UTR (CDK4 WT 3′-UTR) was cloned into the pGL3 vector (pGL3-CDK4 WT 3′-UTR), downstream of the luciferase open reading frame (ORF). In addition, to validate target specificity, we conducted site-directed mutagenesis for CDK4 WT 3′-UTR using the QuikChange Mutagenesis kit in order to destroy the miR-486-5p binding sites (CDK4 mut 3′-UTR, Figure 3A). The relative luciferase activity of the report gene in HEK293T cells cotransfected with pGL3-CDK4 WT 3′-UTR and miR-486-5p mimic was significantly decreased by 30% compared with the control (cotransfected with pGL3-CDK4 WT 3′-UTR and NC mimic). Conversely, co-transfection of miR-486-5p with pGL3-CDK4 mut 3′-UTR resulted in no significant change in luciferase activity, suggesting that miRNA/target 3′-UTR specificity (Figure 3B). We next observed that miR-486-5p reduced both mRNA and protein expression of CDK4 in H1299 and SPCA-1 cells (Figure 3C and 3D).

### CDK4 is upregulated in tumor tissues and cancer cells

**Figure 4:**
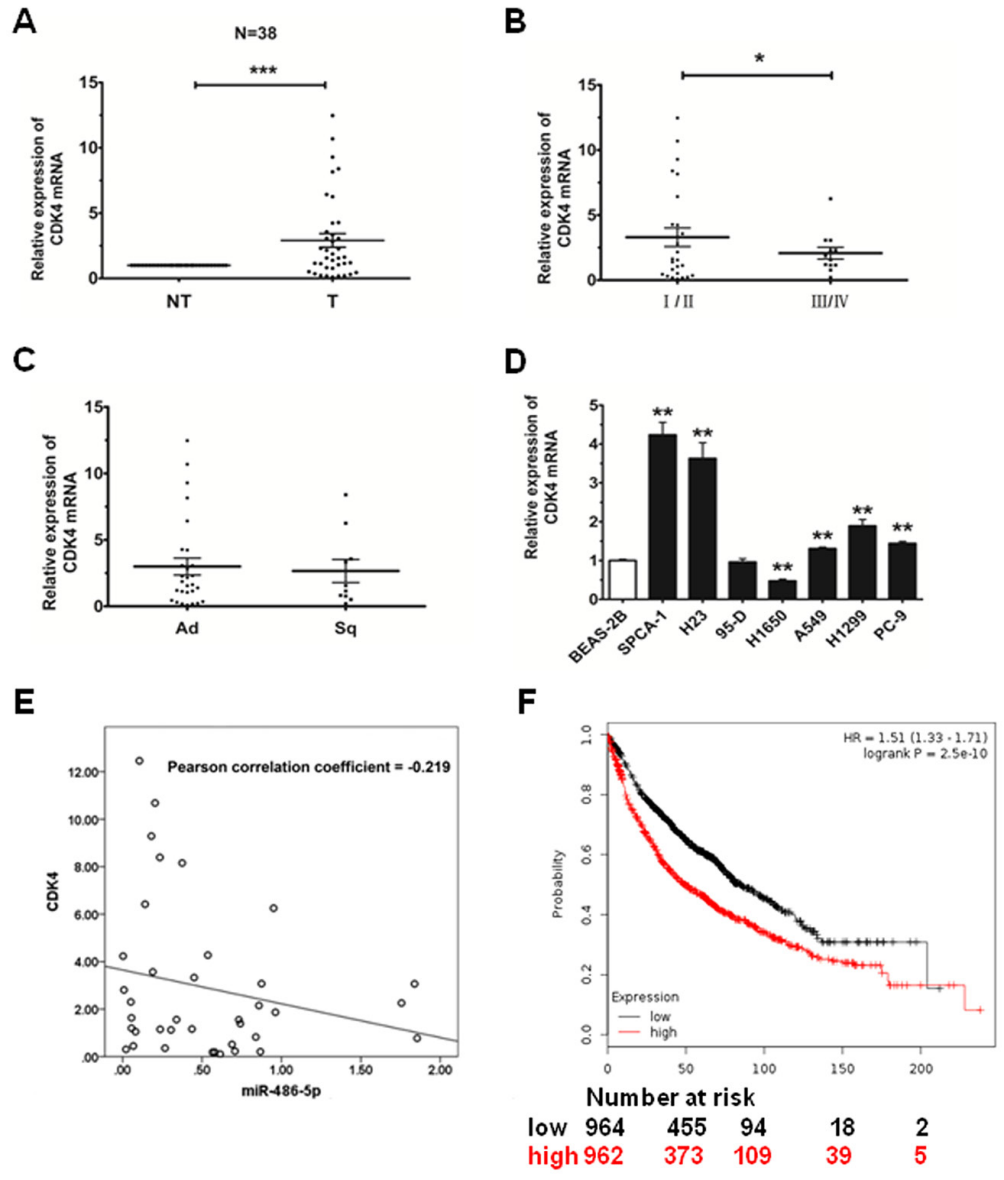
CDK4 is upregulated in NSCLC tissues and cell lines **A.** The relative expression of CDK4 mRNA from qRT-PCR of corresponding non-tumor tissues (NT) and tumor tissues (T). 18S was used for normalization. **B.** The relative expression of CDK4 mRNA in stage I/II tumors and stage III/IV tumors. **C.** The relative expression of CDK4 mRNA in adenocarcinoma (Ad) and squamous cell carcinoma (Sq). **D.** The relative expression of CDK4 mRNA in lung cancer cell lines or a pulmonary epithelial cell line (control) was measured by qRT-PCR. * P<0.05, ** P<0.01, *** P<0.001. **E.** MiR-486-5p had a negative correlation with CDK4 according to Pearson Correlation Coefficient. **F.** The effect of CDK4 expression levels on the overall survival in 1926 lung cancer patients was analyzed and Kaplan-Meier plots were generated using a Kaplan-Meier Plotter (http://www.kmplot.com).

We detected the expression of CDK4 in the same 38 pairs of tissues to determine whether the expression of CDK4 is associated with miR-486-5p in NSCLC. Among the pairs of tissues, we found that the expression level of CDK4 was upregulated in tumor tissues, as compared with their adjacent pair-matched non-tumor tissues (P<0.05, Figure 4A). We observed that the expression of CDK4 was much higher in stage I/II than that in stage III/IV and also showed no significant difference in Ad and Sq (Figure 4B and 4C). We then measured the endogenous expression levels of CDK4 in several NSCLC cell lines, and found that CDK4 expression was much higher in NSCLC cells compared with the BEAS-2B control cells (Figure 4D). Therefore, according to the analysis of Pearson Correlation Coefficient, we surmised that miR-486-5p had a negative correlation with CDK4 (Figure 4E).

Finally, to analyze the effect of CDK4 expression in lung cancer patients, we generated a Kaplan-Meier survival curve of NSCLC patients with low or high expression of CDK4, using the Kaplan-Meier Plotter online database (www.kmplot.com/analysis, Figure 4F). In 1926 cases, we found that NSCLC patients with high expression of CDK4 had lower survival rates.

### Effects of CDK4 siRNA on proliferation and cell cycle in NSCLC cells

**Figure 5:**
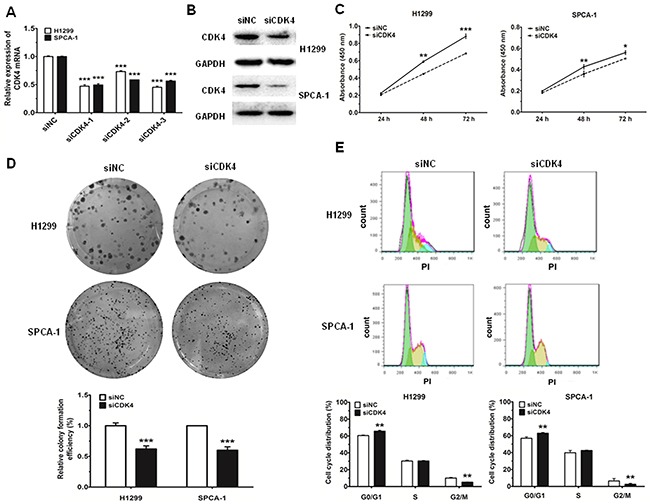
Knockdown of CDK4 can inhibit cell proliferation and impede cell-cycle progression in NSCLC cell lines **A.** and **B.** The mRNA levels of CDK4 were measured by qRT-PCR and the protein levels were measured by western blot and in H1299 and SPCA-1 cells transfected with negative control (siNC) and CDK siRNA (siCDK4). **C.** H1299 and SPCA-1 cells were transfected with siNC or siCDK4, and cell proliferation was determined by CCK-8. **D.** Colony formation assay in H1299 and SPCA-1 cells transfected with siNC or siCDK4. **E.** Cell cycle distributions of H1299 and SPCA-1 cells transfected with siNC or siCDK4 were detected by flow cytometry. * P<0.05, ** P<0.01, *** P<0.001.

To determine the biological effects of miR-486-5p could indeed be attributed to direct targeting of CDK4, we knocked down CDK4 using siRNA and then detected the change in cell proliferation and cell cycle progression. The expression of CDK4 mRNA and protein decreased by more than 50% in cells transfected with siCDK4 after 48 h, as compared to the control (Figure 5A and 5B). Moreover, the results of CCK-8 and cell colony assays showed that the proliferation capacity of NSCLC cells was significantly decreased after treatment with siCDK4 (Figure 5C and 5D). Correspondingly, we also found that, after 48 h of CDK4 knockdown, the proportion of cells at G0/G1 phase increased more than 5% as compared with the control (Figure 5E). Together, these data suggested that the observed effects of miR-486-5p on cell proliferation and cell cycle progression were partially mediated through targeting of CDK4.

### Downregulation of miR-486-5p is due to the hyper-methylation of the miR-486-5p gene promoter region in NSCLC

There are a series of correlations between tumor-suppressor miRNAs and methylation in cancers, as found by Lopez-Serra *et al* [25]. Based on this, we wondered whether the promoter region of miR-486-5p was hypo-methylated in NSCLC.

**Figure 6:**
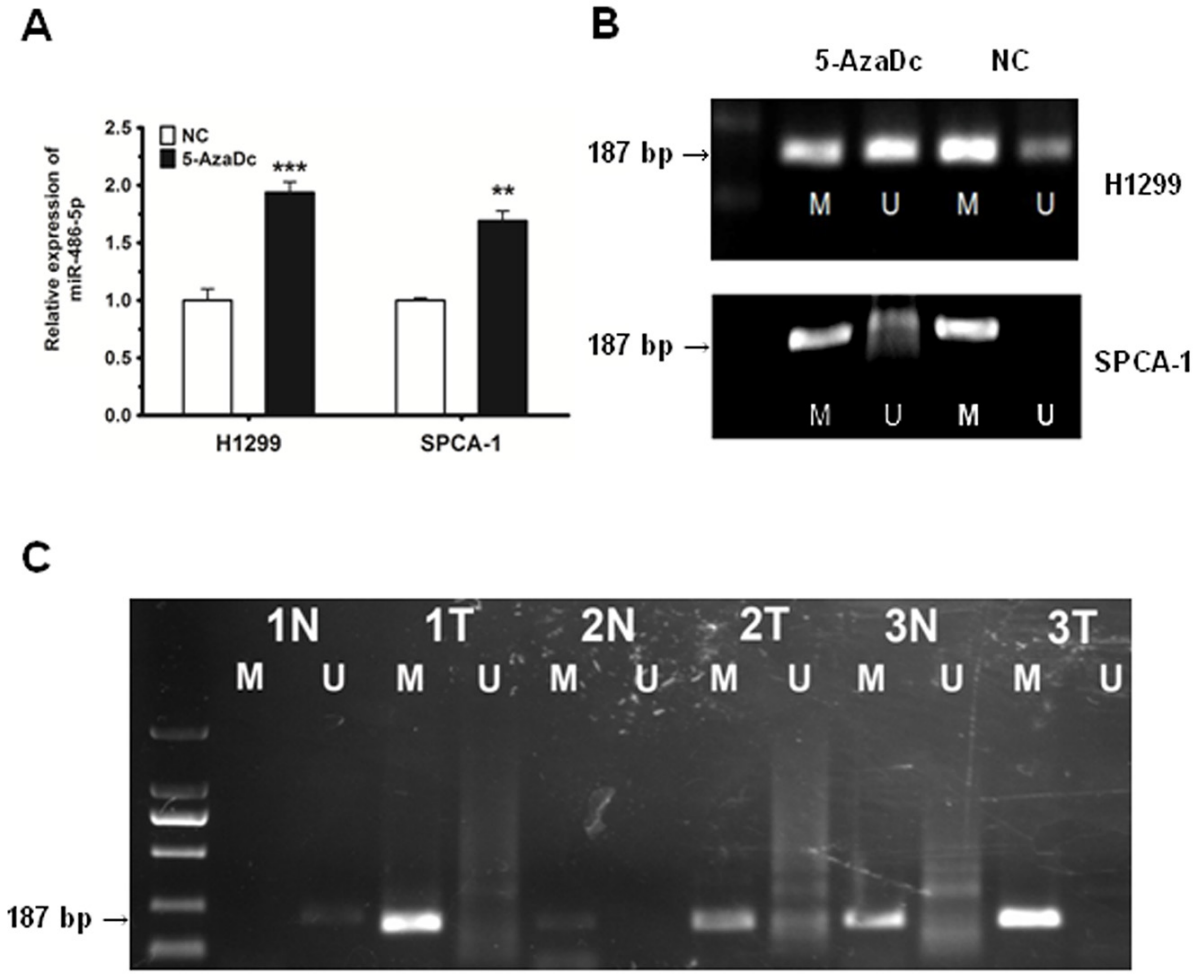
The downregulation of miR-486-5p is due to the hyper-methylation of the miR-486-5p promoter region in NSCLC tissues and cell lines **A.** MiR-486-5p was upregulated in H1299 and SPCA-1 cells after 5 days of 5-AzaDc treatment. **B.** MS-PCR analysis of the miR-486-5p promoter in H1299 and SPCA-1 cells challenged with or without 5-AzaDc for five days. The methylation status of the miR-486-5p promoter region was detected using MS-PCR, where M indicates methylation and U indicates none. **C.** Primer sets used for amplification were designated as methylated (M) or unmethylated (U). Ten μL of PCR product was run on 1% agarose gel, stained with GoldView, and visualized under UV illumination. The numbers displayed indicate pairs of samples, with each pair comprising of one normal tissue (N) and one tumor tissue (T). ** P<0.01, *** P<0.001.

We first observed that following treatment with the DNA demethylating agent 5-aza-2′-deoxycytidine (5-AzaDc), miR-486-5p was strongly upregulated in H1299 and SPCA-1 cancer cell lines (Figure 6A), suggesting that 5-AzaDc acts to induce miR-486-5p expression. In order to ensure that the miR-486-5p promoter was mediated by methylation, we conducted methylation specific PCR (MS-PCR) analysis in H1299 and SPCA-1 cells challenged with or without 5-AzaDc for five days. The results demonstrated that the level of methylation in the promoter region of miR-486-5p was downregulated significantly after the treatment of 5-AzaDc (Figure 6B). We thus concluded that the upstream promoter of miR-486-5p was regulated by methylation in tumor tissues.

Furthermore, we chose to examine 3 pairs of tissues randomly, and detected whether the promoter region of miR-486-5p was regulated by methylation in tumor tissues. Our results revealed that the level of methylation was much higher in these three tumor tissues compared to normal tissues (Figure 6C).

## DISCUSSION

It has been reported that miR-486-5p functions as a tumor suppressor in several cancers [26-28]. Zhang *et al* suggested that miR-486-5p could be a biomarker and play a suppressor gene in NSCLC [19]. Some studies have also shown the effect of miR-486-5p on inhibition of the development and growth of NSCLC in mouse models [29, 30]. However, compared with those papers, we mainly focused on the influence of miR-486-5p on cell cycle in NSCLC, which couldn't be mentioned. In this study, we confirmed that miR-486-5p regulated cell proliferation and cell cycle by directly targeting CDK4. Because CDK4 plays an important role in G1/S phase transition by associating with CDK6 [31-33], deregulation of the CDK4/6 signaling pathway is one of the most common changes found in human cancers [34-36], including NSCLC [37, 38], CDK4/6 were also considered the most desirable targets for cancer therapies [39-41].

Interestingly, in epigenetics, we found that the methylation level of miR-486-5p promoter region CpG islands was significantly up-regulated in NSCLC tissues compared with that in normal tissues. It means the expression of miR-486-5p affected NSCLC by methylation. The expression of miR-486-5p was remarkably different in early and advanced TNM stages. Higher expression of miR-486-5p was seen in early stages, while lower expression in advanced stages in our cases. We speculated that miR-486-5p played important roles in tumorgenesis, and more complicated factors might be involved in advanced in NSCLC in advance stages. These results suggested that miR-486-5p could be used as a possible diagnostic marker for early stages.

In summary, our study focused on the mechanism of miR-486-5p as a tumor suppressor in NSCLC, and our findings revealed that it functioned, in part, through targeting CDK4, an important component of the CDK4/6 signaling pathway. Our data supported the hypothesis that re-introduction of miR-486-5p into NSCLC cells could reduce the expression of the oncogenic target gene, an encouraging finding which suggests that miRNAs could be potential drug targets for the treatment of NSCLC. A separate study reported that miR-486-5p inhibited cell growth in a series of tumors [16, 26, 27, 29, 42], which substantiates the concept that miR-486-5p may be less tumor specific than other miRNAs, making it a more dependable therapeutic strategy. In some papers published, only several genes have been proven to be direct genes of miR-486-5p, including IGF1R and ARHGAP5 [29, 43], and CDK4 also plays an important role in some pathways in NSCLC. As a result of above, the relationship between miR-486-5p and CDK4 is greatly beneficial to the treatments of cancers through interacting with other miRNAs and genes, and it is valuable to make further studies concerned with miR-486-5p or CDK4.

**Figure 7:**
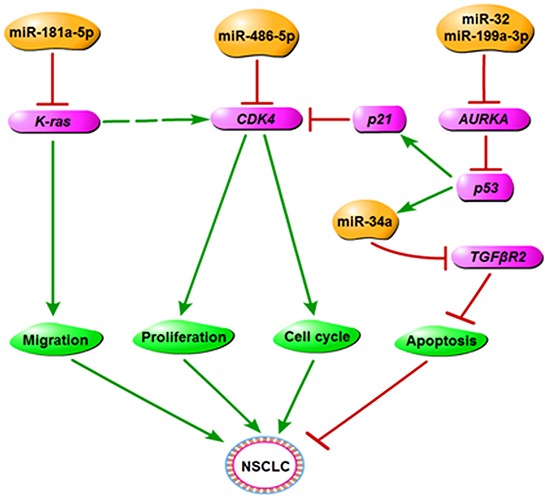
The regulatory network of miR-486-5p in NSCLC We propose that miR-486-5p plays the role of tumor suppressor in NSCLC by downregulating CDK4 and has a close correlation with other miRNAs or genes that are related to the cancer process.

Overall, based on this and prior studies in our lab [8, 13, 14], we concluded that miR-486-5p could be involved in the CDK4/6 signaling pathway by directly targeting CDK4, as well as have a close correlation with the Kras and NF-κB signaling pathway, some miRNAs related these pathways are investigated in our group. In this regulation network, miRNAs can affect the migration, proliferation, apoptosis, and cell cycle of NSCLC (Figure 7). Our studies also found Chinese traditional medicine, such as tanshinones, can regulate miRNAs and corresponding targets to suppress NSCLC [14], and especially some miRNAs can improve positive effects on diabetes. Our findings have potential for us to understand the mechanism of oncogenesis of lung cancer by miRNAs, and drug targets could be used in diagnosis and therapeutic treatment in future.

## MATERIALS AND METHODS

### Cell culture

The human lung epithelial cell line BEAS-2B and the human NSCLC cell lines A549, H1650, PC-9, 95-D and SPCA-1 were obtained from the Cell Bank, China Academy of Sciences (Shanghai, China). H1299 and H23were obtained from the American Type Culture Collection (ATCC, Manassas, USA). A549, PC-9, 95-D, and SPCA-1 were cultured in DMEM medium (Corning Cellgro, Manassas, USA) supplemented with 10% (v/v) fetal bovine serum (FBS, Gibco, Gaithersburg, USA). H1299, H23, and H1650 were cultured in RPMI 1640 medium (Corning Cellgro) supplemented with 10% (v/v) FBS (Gibco). BEAS-2B was cultured in LHC medium (Gibco) supplemented with 10% (v/v) FBS (Gibco). All cells were cultured at 37°C in 5% CO_2_ atmosphere.

### Tissue samples

The tissue samples were all obtained from the Shanghai Chest Hospital affiliated with Shanghai Jiao Tong University with approval from the ethics committee of Shanghai Chest Hospital. All the details of samples used in this paper were listed in Supplementary Table S1

### Transfection

H1299 and SPCA-1 cells were transiently transfected with 100 nM of miR-486-5p mimic, negative control mimic (NC), CDK4 siRNA (siCDK4), or negative control siRNA (siNC) (RIBOBIO, Guangzhou, China) using Invitrogen™ Lipofectamine 2000 (Life Technologies, New York, USA) according to the manufacturer's recommendations. After 24 to 48 h post-transfection, cells were used for subsequent experiments including assays for proliferation and cell cycle analysis.

### RNA isolation, reverse transcription, and quantitative real-time PCR (qRT-PCR)

Total RNA was isolated using Trizol Reagent (Sangon Biotech, Shanghai, China) and cDNA synthesis was performed with the SYBR^®^ PrimeScript™miRNA RT-PCR Kit and PrimeScript™ RT Master Mix (Takara Biotech, Otsu, Japan) following the manufacturer's instructions for each reagent or kit. MiRNA and mRNA analyses were performed by qRT-PCR using SYBR Green II (Takara Biotech) according to manufacturer's protocol, with a CFX96^TM^ Real-time System (Bio-Rad, California, USA). Relative quantification of miR-486-5p was obtained by normalization to U6 expression levels, and relative quantification of CDK4 was obtained by normalized to 18S rRNA expression levels. The expression levels of mRNAs and miRNAs were determined by the 2^-ΔΔCt^ method for relative quantification of gene expression. ΔCt and ΔΔCt were calculated using the following formulae: ΔCt = Ct_miR-486-5p_- Ct_U6_ or Ct_CDK4_ - Ct_18S_ and ΔΔCt = ΔCt_case_ - ΔCt_control_.

### Cell proliferation analysis

Cell proliferation was measured with the Cell Counting Kit-8 (CCK-8) assay kit (Dojindo, Tokyo, Japan). Six hours after transfection, cells were plated in a 96-well microplate (Corning Incorporated, New York, USA) and incubated at 37°C in 5% CO_2_. Each data point was measured from 3 replicate wells. After 24, 48, and 72 h of culture, 8 μl of CCK-8 solution was added to each well with 100 μl serum free medium and incubated for 90 min. The absorbance was then measured at 450 nm by a multi-function enzyme-linked analyzer, FLx8 (BioTek, Vermont, USA).

### Colony formation assay

Cells were transfected with miR-486-5p mimic or CDK4 siRNA. Twenty-four hours later, miR-486-5p expressing cells and CDK4 siRNA cells were trypsinized, counted, plated in 3 cm dishes at a density of 500 cells, and then cultured at 37°C in 5% CO_2_ atmosphere. Ten days later, colonies resulting from the surviving cells were fixed with 300 μL of methanol for 15 min, stained with 300 μL crystal violet for 15 min, and counted.

### Cell cycle analysis

Cell cycle distribution was assessed by propidium iodide (PI) staining. Treated cells (1×10^5^ cells) were harvested and fixed with 75% ethanol at −20°C overnight. Cells were washed twice with PBS and then resuspended in 250 μL of RNase A buffer (100 ng/mL) at room temperature for 30 min. After this step, a 2′ solution of PI (100 ng/mL) was added to the mixture which was then incubated for 15 min in the dark, followed by filtrating with 200 mesh filter membrane. Results were determined by analysis on the MoFlo XDP flow cytometer sorting system (Beckman Coulter, Inc., Brea, USA).

### Dual luciferase reporter assay

The CDK4 WT 3′-UTR firefly luciferase construct (pGL3-CDK4 WT 3′-UTR) was generated by inserting a 683 bp fragment of human CDK4 3′-UTR into the *Xba* I /*Eco*R I sites of the pGL3 Luciferase Report vector. The pGL3-CDK4 mut 3′-UTR construct was generated by mutation of the complementary seed sequence to the miR-486-5p binding region. HEK293T cells were co-transfected with 150 ng pGL3-CDK4 WT 3′- UTR or pGL3-CDK4 mut 3′-UTR luciferase reporter and 15 ng Renilla luciferase reporter (pRL) using Invitrogen™ Lipofectamine 2000 (Life Technologies, New York, USA). Cells were incubated for 48 h and luciferase activity was assayed by an Orion II Microplate Illuminometer (Titertek-Berthold, South San Francisco, USA) according to the manufacturer's instructions. Firefly luciferase units were normalized against Renilla luciferase units to control for transfection efficiency, and relative activities were expressed as the fold-change in luciferase activity. All the primers used in this paper were listed in Supplementary Table S2.

### Western blot analysis

Total protein was extracted using RIPA lysis buffer (CWBIO, Beijing, China) and quantified by Bradford assay [44]. Equal amounts of protein from each sample were subjected to SDS-PAGE electrophoresis and transferred to a polyvinylidene fluoride (PVDF) membrane (Millipore Corporation, Billerica, USA). The membrane was then soaked in tris-buffered saline with Tween-20 (TBST, 150 mM NaCl, 20 mM Tris-HCl pH 8.0, 0.05% Tween-20) containing 5% bovine serum albumin (BSA) for 1 h at room temperature, followed by gentle shaking and subsequent incubation with specific antibody against CDK4 or GAPDH (1:1000, Cell Signaling Technology, Danvers, USA) at 4°C overnight. Afterwards, the membrane was washed and incubated with a horseradish peroxidase (HRP)-conjugated secondary antibody (1:10000, Signalway Antibody, Nanjing, China) for 1 h at room temperature. Protein bands were detected using a chemiluminescent HRP substrate (Millipore Corporation, Billerica, USA) and analyzed by Image Lab analysis software (Bio-Rad). CDK4 was normalized to GAPDH and expressed as a percentage of control.

### 5-aza-2′-deoxycytidine treatment

H1299 and SPCA-1 cells were seeded in 6 cm culture dishes, and allowed to reach a density of 50% at which point they were treated with 5-aza-2′-deoxycytidine (5-AzaDc, Sigma-Aldrich, Missouri, USA). Fresh medium and 5-AzaDc were added every 24 h until completion of the 3-day treatment.

### Mthylation-specific PCR (MS-PCR)

Following the protocol published by Gipsy Majumdar, G. *et al* [45], genomic DNA from tissues and cell lines was isolated with Trizol Reagent (Sangon Biotech). A bisulfite treatment was conducted using the EpiTect Bisulfite Kit (Qiagen, Duesseldorf, Germany) according to the protocol published by Long, X.R. *et al* [46]. PCR was first performed on purified DNA using methylated primers and unmethylated primers under the following conditions: 95°C for 3 min, followed by 30 cycles of 95°C for 30 sec, 50°C (for unmethylated) or 53°C (for methylated) for 30 sec, 72°C for 1 min, and a final extension at 72°C for 5 min. Then, 15 μL of PCR product was detected by agarose gel electrophoresis using 1% agarose gel stained with GoldView. To ensure reproducibility, the MS PCRs were performed three times. The methylated and unmethylated primers used in this paper were listed in Supplementary Table S2.

### Statistical analysis

Statistical analysis was performed using SPSS v.19.0 software and graph presentation was completed using GraphPad Prism 5 Software. Results are represented as the mean ± SEM, and the difference between two experimental groups was evaluated using student's t-test with statistical significance defined as P<0.05.

## SUPPLEMENTARY TABLES


