# Supplementary material for: Direct repression of the oncogene CDK4 by the tumor suppressor miR-486-5p in non-small cell lung cancer
Source: Oncotarget. 2016 Mar 31;7(23):34011–21. doi: 10.18632/oncotarget.8514 (PMC5085134; doi:10.18632/oncotarget.8514)
Supplement: Supplementary file 1 [file oncotarget-07-34011-s001.pdf]

SUPPLEMENTARY TABLES

Supplementary Table S1: Descriptive characteristics of samples

Characteristic	Adenocarcinoma (n=28)	Squamous Cell carcinoma (n=10)
Median age (range)	65 (47-78)	65 (53-71)
Gender		
Male	16 (50%)	9 (90%)
Female	12 (50%)	1 (10%)
Stage, n (%)		
I A	6 (21.4%)	3 (30%)
I B	8 (28.6%)	1 (10%)
II A	3 (10.7%)	2 (20%)
II B	2 (7.1%)	1 (10%)
III A	6 (21.4%)	2 (20%)
III B	0 (0%)	1 (10%)
IV	3 (10.7%)	0 (0%)

Supplementary Table S2: PCR primers

Primer Name	Primer Sequences
Methylated Primer	Forward: GGTAGTTAGGATTCGGAGAC<br>Reverse: GAAAACTAAACCAACGACGT
Unmethylated Primer	Forward: GAGGTAGTTAGGAGTTTGGAGAT<br>Reverse: CAAAAAACTAAACCAACAACATA
CDK4 WT 3'-UTR	Forward: GCTCTAGAGCCATTTCCCTTCTGGACACTG<br>Reverse: GGAATTCATCTCGGCTCACCGCAACCT
CDK4 mut 3'-UTR	Forward: ATATTTGGGGTCCTTTTTTACATATGAAAAA CAAAACAAAGAAATAATGG<br>Reverse: CCATTATTTCTTTGTTTTGTTTTTCATATGTAAAAAAG GACCCCAAATAT
